# Journey through tumorverse: Creating models to decode PXA mysteries

**DOI:** 10.1016/j.omton.2024.200853

**Published:** 2024-08-06

**Authors:** George Bukenya, Anthony R. Sloan, Justin D. Lathia

**Affiliations:** 1 Lerner Research Institute, Cleveland Clinic, Cleveland, OH, USA; 2 Case Comprehensive Cancer Center, Cleveland, OH, USA; 3 Rose Ella Burkhardt Brain Tumor & Neuro-Oncology Center, Cleveland Clinic, Cleveland, OH, USA

## Abstract

Pleomorphic xanthoastrocytoma (PXA) is a rare pediatric low-grade glioma (pLGG), of which 60%–80% exhibit the BRAF V600E mutation, that enhances the aggressiveness and progression to an eventual pediatric high-grade glioma (pHGG). Despite the aggressiveness of this mutational status, the mechanisms underlying the progression of BRAF V600E tumors remain poorly understood, primarily due to limited insights into their *in vivo* growth dynamics. In this issue, Rajappa and colleagues leverage a novel immunocompetent RCAS-BRAF V600E murine glioma model to profile the immunological dynamics taking place in BRAF V600E pLGG.

## Main text

Pediatric low-grade gliomas (pLGGs) are among the most prevalent pediatric brain tumor, accounting for 35% of all central nervous system tumors.^1^ Genetically, they commonly exhibit alterations in the MAPK/ERK activation pathway, which are not typically seen in adult LGGs.^1^ Regarding treatment, patients with pLGG are oftentimes tumor free following surgery.^2^ Of the pediatric patients diagnosed with pleomorphic xanthoastrocytoma (PXA), 60%–70% will carry the BRAF V600E mutation. This mutational status often correlates with progression into more aggressive, high-grade gliomas (HGGs) and subsequent worse overall survival. Studying the characteristics of pLGGs, specifically the tumors with the V600E mutational status, pose significant challenges due to the lack of adequate models to successfully study the low-grade to high-grade transition of these tumors.

Previous studies have extensively investigated immune cell infiltration in adult glioblastoma (GBM) tumors and identified distinct immune cell profiles, categorizing them into three groups: (1) tumors with leukocyte infiltration, (2) tumors enriched with tumor-associated macrophages (TAMs) and neutrophils, and (3) tumors exhibiting a mixed infiltration of TAMs, neutrophils, and T lymphocytes.^3^ These findings underscore the heterogeneity in immune cell infiltration across GBM tumors. While the BRAF V600E mutation is recognized as a key driver of tumor progression in pLGGs, how these tumors regulate the immune cell infiltration to drive their progression remains underexplored due to adequate models.

It is important to recognize that mouse models provide invaluable insights into the mechanisms of disease and the efficacy of potential therapies. The RCAS-TVA system is a commonly used model that utilizes viral vectors to deliver gene-editing proteins, inducing specific mutations to initiate tumors in mice with known glioma oncogenic drivers. Other *in vivo* models include carcinogen-induced tumors, xenografts of tumor cell lines, and genetically engineered rodent modes.^4^ Although these models offer the advantage of developing tumors *de novo* and maintaining tumor-host interactions, they often produce circumscribed tumors, lack the complex heterogeneity seen clinically, and do not thoroughly give insight into the low-grade to high-grade disease course. The RCAS-BRAF V600E model developed by Rajappa and colleagues represents a significant advancement, providing a valuable tool for exploring the role of immune cell populations in the progression of V600E pLGG to its lethal HGG. The BRAF V600E mutation has been identified in multiple cancers including thyroid, ovarian, lung, and melanoma. BRAF, activated by GTPase proteins, dimerizes—either as homodimers or heterodimers—to activate the MAPK pathway, including MEK1 and MEK2, through phosphorylation.^5^ Pathologically, the BRAF mutation is perpetually activated due to point mutations, fusions, or in-frame deletions.^5^

**Figure 1:**
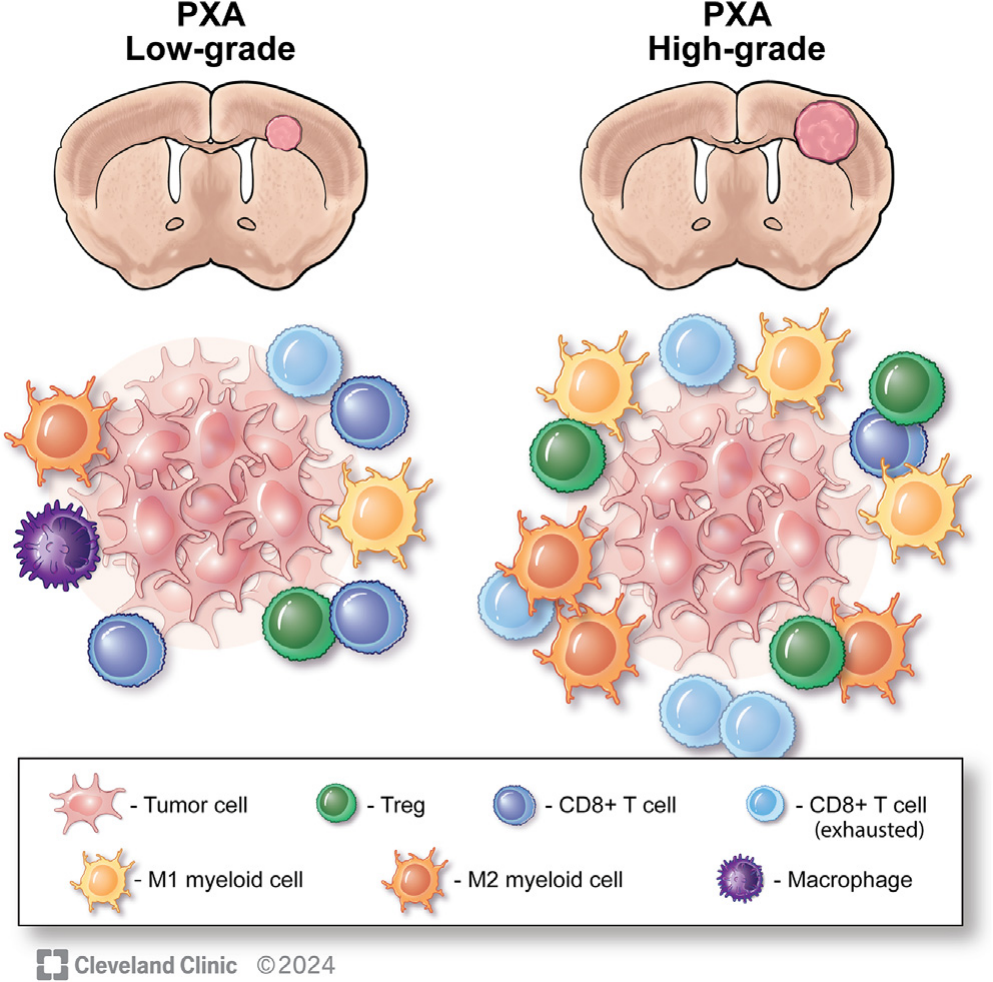
Summary of findings by Rajappa and colleague utilizing their novel PXA BRAF V600E model

To further elucidate the characteristics of PXA pLGGs, Rajappa and colleagues successfully initiated gliomagenesis, replicating the histological, pathological, and morphological features observed in BRAF V600E PXA. This model will continue to be valuable in investigating the transition from LGGs to HGGs in the context of BRAF V600E mutations. Specifically, this model will allow a mechanistic understanding of immune cell infiltration patterns that take place during the low-grade to high-grade transition. Utilizing this model, Rajappa and colleagues conducted an in-depth analysis of the tumor microenvironment and immune cell infiltration at the end stage (day 50) of their new model. RNA sequencing revealed 8,085 genes with significant differential expression compared to control animals. Notably, there was an upregulation of genes linked to CD8+ T cell activation and exhaustion, as well as both proinflammatory (M1) and immunosuppressive (M2) myeloid cells, antigen presentation, and regulatory T cell activation, highlighting the roles of cytotoxic T cell exhaustion and myeloid cell immunosuppression in the low-grade to high-grade transition (Figure 1).

Limitations with the RCAS-BRAF V600E model primarily revolve around its median survival time, which is notably short at 46 days. This constraint significantly hampers the scope of pharmacological studies involving BRAF inhibitors, as the limited survival period reduces the window for meaningful intervention and observation. In addition, the rate at which this model progresses from a low-grade to high-grade tumor is variable. Additional limitations of this study stem from the inherent species differences between mouse and human biology, which may affect the translatability of the findings. The tumor heterogeneity observed in human pediatric gliomas may not be fully replicated by the murine model, thereby limiting the model’s ability to capture the complexity of the disease’s pathology. Moreover, the model’s restricted scope of mutations does not reflect the diverse and often concurrent genetic alterations characteristic of pediatric gliomas.

To enhance the utility of this model, it is crucial to delve deeper into several key areas. Firstly, quantifying immune cell infiltration at various stages of disease progression is essential to elucidate the dynamics of the tumor microenvironment and immune cell interactions as well as give a clear understanding of the dynamic disease progression. Secondly, understanding the median survival and low-grade to high-grade transition with respect to sex as a biological variable could yield insights into sex-specific responses and survivability, potentially informing personalized therapeutic approaches. Additionally, it is important to explore the translational potential of this model in the development of BRAF inhibitors for *in vivo* studies of pLGGs and conduct further testing for potential combinational therapies in pLGGs. Another critical area of exploration is the mechanisms of tumor immune evasion, which could reveal novel targets for immunotherapy. Furthermore, a comprehensive understanding of tumor-host interactions and the intricacies of tumor formation is imperative to ensure that the RCAS-BRAF V600E model accurately mirrors the pathological and biological characteristics of human tumors. With ongoing research and advancements in this area, the RCAS-BRAF V600E model holds great promise for enhancing our understanding of human tumors and paving the way for innovative therapeutic strategies.
